# IMMEDIATE AND FUTURE IMPLICATIONS

**DOI:** 10.1093/geroni/igac059.926

**Published:** 2022-12-20

**Authors:** Martha Williman

**Affiliations:** Ohio Council for Cognitive Health, Findlay, Ohio, United States

## Abstract

ODRC leadership was engaged in developing and promoting this first-of-its-kind initiative. Success was enhanced by making the training mandatory and providing CEs for employees in ODRC’s medical, behavioral health, mental health, dental and dietary departments. Our goals are to expand to all the ODRC’s over 12,000 employees and to increase dementia education in correctional settings across the U.S. In addition, we propose further developing the program to include people living in incarcerated settings and those being released into the community. With 95% of staff agreeing they would be able to use what they learned in their workplace, and over 63% of attendees stating they know someone who lives/lived with dementia, this training has implications for not only professional development, but personal support as well and provides a starting point for future education sessions to serve both staff and those living in incarcerated settings within and beyond Ohio.

